# SARS-CoV-2 detection in pediatric dental clinic wastewater reflects the number of local COVID-19 cases in children under 10 years old

**DOI:** 10.1038/s41598-024-63020-z

**Published:** 2024-05-28

**Authors:** Dai Kanamori, Jun Sakai, Takahiro Iijima, Yuka Oono, Bikash Malla, Eiji Haramoto, Satoshi Hayakawa, Shihoko Komine-Aizawa, Shigefumi Maesaki, Thomas Vorup-Jensen, Paul Evan Kilgore, Hikaru Kohase, Tomonori Hoshino, Mitsuko Seki

**Affiliations:** 1 Division of Pediatric Dentistry, Department of Human Development and Fostering, Meikai University School of Dentistry, 1-1 Keyakidai, Sakado, Saitama 350-0283 Japan; 2 Department of Infectious Disease and Infection Control, Saitama Medical University, Saitama, 350-0495 Japan; 3 Division of Dental Anesthesiology, Department of Diagnostic and Therapeutic Sciences, Meikai University School of Dentistry, Saitama, 350-0283 Japan; 4 Interdisciplinary Center for River Basin Environment, University of Yamanashi, Yamanashi, 400-8511 Japan; 5 Division of Microbiology, Department of Pathology and Microbiology, Nihon University School of Medicine, Tokyo, 173-8610 Japan; 6 Biophysical Immunology Laboratory, Department of Biomedicine, Aarhus University, 8000 Aarhus C, Denmark; 7 Department of Pharmacy Practice, Eugene Applebaum College of Pharmacy and Health Sciences, Wayne State University, Detroit, MI 48201 USA

**Keywords:** Water microbiology, Vaccines, Virology, Viral infection, Epidemiology, Paediatric research, Risk factors, Epidemiology, Population screening, Paediatrics, Paediatric research

## Abstract

This was the first longitudinal study to analyze dental clinic wastewater to estimate asymptomatic SARS-CoV-2 infection trends in children. We monitored wastewater over a 14-month period, spanning three major COVID-19 waves driven by the Alpha, Delta, and Omicron variants. Each Saturday, wastewater was sampled at the Pediatric Dental Clinic of the only dental hospital in Japan’s Saitama Prefecture. The relationship between the weekly number of cases in Saitama Prefecture among residents aged < 10 years (exposure) and wastewater SARS-CoV-2 RNA detection (outcome) was examined. The number of cases was significantly associated with wastewater SARS-CoV-2 RNA positivity (risk ratio, 5.36; 95% confidence interval, 1.72–16.67; Fisher’s exact test, *p* = 0.0005). A sample from Week 8 of 2022 harbored the Omicron variant. Compared to sporadic individual testing, this approach allows continuous population-level surveillance, which is less affected by healthcare seeking and test availability. Since wastewater from pediatric dental clinics originates from the oral cavities of asymptomatic children, such testing can provide important information regarding asymptomatic COVID-19 in children, complementing clinical pediatric data.

## Introduction

Coronavirus disease 2019 (COVID-19) has continued to spread since the World Health Organization (WHO) declared its pandemic in March 2020. During the first phase of the pandemic, infections in children were rare. However, the emergence of the Delta and Omicron variants led to an increase in the number of pediatric cases^1^. Despite an increasing number of children with COVID-19, as well as occasional reports of severe disease and disease spread from children, vaccination coverage among children remains lower than in older individuals^1^. In children, COVID-19 can cause severe illness, particularly in those with underlying medical conditions; however, children without underlying medical conditions can also experience severe illness^2^. Rarely, some infected children can later develop multisystem inflammatory syndrome in children (MIS-C)^3^. However, little information is available on asymptomatic COVID-19 infection. It is important to clarify the situation of asymptomatic children to prevent the spread of infection and development of severe illness in children.

In May 2023, the Japanese government switched COVID-19 monitoring from notifiable disease surveillance to sentinel surveillance^4^. It is difficult to survey large numbers of people regularly, and vaccine usage made surveillance of only symptomatic patients inaccurate because the number of asymptomatic carriers increased. During the pandemic, technologies to detect the virus in wastewater were developed, and wastewater became an efficient community sample source. Both asymptomatic and symptomatic individuals infected with SARS-CoV-2 shed the virus in their feces. Detection of SARS-CoV-2 in wastewater provides information on the prevalence of COVID-19 in a community^5^ and can provide data for communities where timely COVID-19 clinical testing is underused or unavailable. To capture the population dynamics of COVID-19, wastewater surveillance was extended to multiple sites in Japan^6–10^.

General wastewater originates from households and buildings (such as toilets, showers, and sinks) and may contain human fecal waste as well as water from non-household sources (such as rain and industrial use).　Wastewater from pediatric dental clinics has a relatively restricted origin. During the COVID-19 pandemic, saliva and other viral-RNA-rich fluids^11^ discharged from the oral cavity of subclinically infected children. Symptomatic patients do not receive dental treatment. Compared to general community wastewater, wastewater from pediatric dental clinics can provide a targeted sample of asymptomatic children, and offer information on the status of children with asymptomatic COVID-19. A few studies reported on SARS-CoV-2 RNA in wastewater from schools^12,13^, and one study from Scotland examined polymerase chain reaction (PCR) results from individual dental patients, although it was limited to a 13-week period^14^. However, no previous study has reported on SARS-CoV-2 RNA in dental wastewater, and it is not known how this may relate to the infection status of the local community.

In this prospective longitudinal observational study, we hypothesized that SARS-CoV-2 in the wastewater from a pediatric dental clinic, originating from the oral cavities of asymptomatic children, would be related to the number of local pediatric COVID-19 cases. We compared the number of weekly reported new cases (NWRNC) in the local pediatric population with the detection results.

## Results

**Figure 1:**
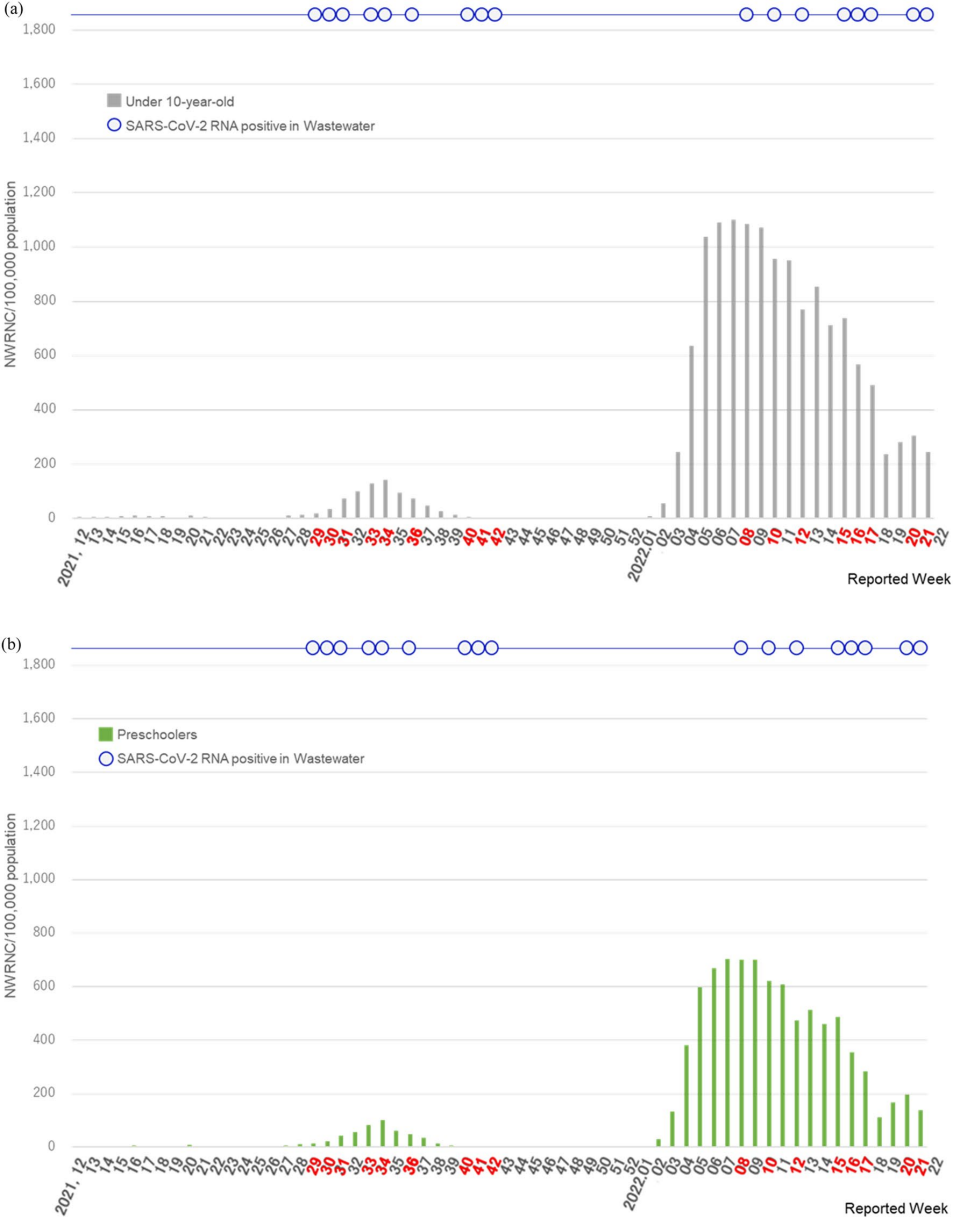

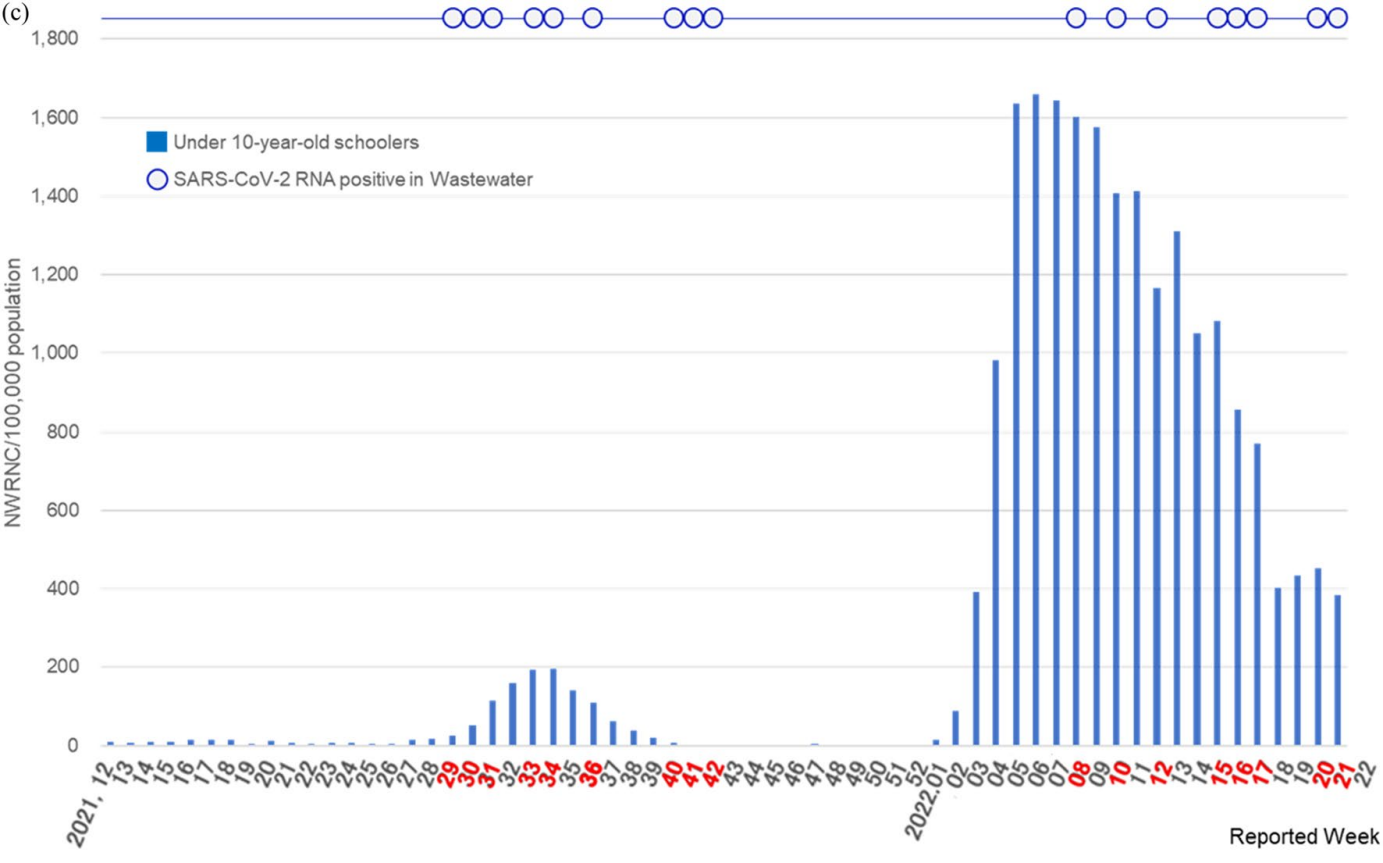
Weekly detection of SARS-CoV-2 RNA in wastewater from a pediatric dental clinic and number of weekly reported new cases (NWRNC) per 100,000 population in Saitama Prefecture, Japan, in total < 10-year-olds (**a**), preschoolers (**b**), and schoolers aged < 10-year-old (c). SARS-CoV-2 RNA detection was observed during nine weeks in 2021 (Weeks 29, 30, 31, 33, 34, 36, 40, 41, and 42) and eight weeks in 2022 (Weeks 8, 10, 12, 15, 16, 17, 20, and 21), highlighted in red text.

In Japan’s Saitama Prefecture (Supplementary Fig. S1), among the 571,180 children aged < 10 years (pre-schoolers, n = 328,311; schoolers, n = 242,869; Table 1) analyzed between Week 12 of 2021 and Week 21 of 2022 (wastewater sampling failed in Week 11 of 2021, as stated below), the cumulative number of confirmed COVID-19 cases was 81,780 (preschoolers, n = 29,287; schoolers, n = 52,493; Supplementary Table S1). The median NWRNC was 75.0 (interquartile range [IQR]: 17.75–1638.00), with values of 33.5 among preschoolers (IQR: 7.50–574.00) and 44.5 among schoolers (IQR: 11.75–1064.00). The cumulative number of confirmed COVID-19 cases per 100,000 individuals during the survey period was 14,317.7 (preschoolers, n = 8920.5; schoolers, n = 21,613.7; Supplementary Table S1), and the median NWRNC was 13.1 (IQR: 3.11–286.77), with a value of 10.2 among preschoolers (IQR: 2.28–174.83) and 18.3 among schoolers (IQR: 4.84–438.10). The NWRNC/100,000 population was significantly higher among schoolers than among preschoolers (*p* < 0.0001, Wilcoxon matched-pairs test), and there was a strong correlation between the NWRNC/100,000 population in schoolers and preschoolers (Spearman’s correlation coefficient [r_s_] = 0.9628, *p* < 0.0001) (Fig. 1).

**Table 1:** Risk of COVID-19 emergence-associated SARS-CoV-2 RNA detection in wastewater from a pediatric dental clinic in Saitama Prefecture, Japan, by age group.

	NWRNC/100,000 population**	SARS-CoV-2 RNA detection trials (n = 58)		RR (95% CI); *p* value^†^
		Yes (n = 17)***	No (n = 41)	
Total, under 10 Y-olds (n = 571,180)	–	–	–	5.36 (1.72–16.67); *p* = 0.0005
NWRNC* < 107 (n = 31)	< 18.7 (n = 31)	3 (9.7%)	28 (90.3%)	–
≥ 107 (n = 27)	≥ 18.7 (n = 27)	14 (51.9%)	13 (48.1%)	–
Preschoolers (n = 328,311)	–	–	–	5.36 (1.72–16.67); *p* = 0.0005
NWRNC < 44 (n = 31)	< 13.4 (n = 31)	3 (9.7%)	28 (90.3%)	–
≥ 44 (n = 27)	≥ 13.4 (n = 27)	14 (51.9%)	13 (48.1%)	–
Schoolers (n = 242,869)	–	–	–	5.36 (1.72–16.67); *p* = 0.0005
NWRNC < 63 (n = 31)	< 25.9 (n = 31)	3 (9.7%)	28 (90.3%)	–
≥ 63 (n = 27)	≥ 25.9 (n = 27)	14 (51.9%)	13 (48.1%)	–

Data are n (%) or RR (95% CI). RR, risk ratio.

*NWRNC, number of weekly reported new cases.

**The cut-off points of NWRNC per 100,000 population were determined based on ROC curve (Supplementary Table S4).

***SARS-CoV-2 RNA detection was observed in 17 weeks (Weeks 29, 30, 31, 33, 34, 36, 40, 41, and 42 in 2021; Weeks 8, 10, 12, 15, 16, 17, 20, and 21 in 2022).

^†^*P*-values obtained by Fisher’s exact test.

The survey period encompassed three COVID-19 waves, characterized by different variants of concern (VOCs): the fourth (Alpha variant), fifth (Delta variant), and sixth (Omicron variant) epidemic waves. The fourth wave had little effect on children aged < 10 years in Saitama Prefecture. Therefore, only the fifth and sixth waves of the epidemic (Weeks 29–39 of 2021 and Weeks 3–21 of 2022, respectively) were observed (Fig. 1).

A total of 9,689 patients underwent dental treatment at the pediatric dental clinic during the survey period (Supplementary Table S2). The SARS-CoV-2 RNA detection results in dental wastewater are shown in Fig. 1 and Supplementary Table S3. Two of the sixty samples were excluded because of failures in the first sampling (Week 11 of 2021) and in the detection of process control (Week 13 of 2022), respectively. Thus, 58 samples were analyzed (n = 58/60, 96.7%). Wastewater samples were positive for SARS-CoV-2 RNA during 9 weeks in 2021 (Weeks 29, 30, 31, 33, 34, 36, 40, 41, and 42) and 8 weeks in 2022 (Weeks 8, 10, 12, 15, 16, 17, 20, and 21). A total of 17 samples were positive (n = 17/58, 29.3%), while 41 were negative (n = 41/58, 70.7%). Only the G339D SARS-CoV-2 mutation (Omicron VOC) was detected in Week 8 of 2022; N501Y and L452R were not detected (Supplementary Table S3).

Concordant with the NWRNC among children aged < 10 years in Saitama Prefecture, SARS-CoV-2 RNA was detected in dental wastewater during the fifth and sixth waves of the COVID-19 pandemic, but not during the period of Alpha variant predominance (Fig. 1). The Delta and Omicron waves (Weeks 29–39 of 2021 and Weeks 3–21 of 2022) tended to precede SARS-CoV-2 RNA detection in dental wastewater (Weeks 29–42 of 2021 and Weeks 8–21 of 2022).

The association between the NWRNC per 100,000 population in Saitama Prefecture (exposure) and the positivity of SARS-CoV-2 RNA in wastewater from a pediatric dental clinic (outcome) was calculated. This was performed after obtaining the optimal cut-off values through receiver operating characteristic (ROC) curve analysis and determining the area under the ROC curve (AUC), as shown in Table 1 and Supplementary Table S4. Among Saitama Prefecture residents aged < 10 years, the risk of COVID-19 epidemic-associated SARS-CoV-2 RNA detection in dental wastewater was 9.7% (n = 3/31) when the NWRNC/100,000 population was < 18.7, and it was 51.9% (n = 14/27) when NWRNC/100,000 population was ≥ 18.7 (Table 1). Compared to all children aged < 10 years, preschoolers and schoolers aged < 10 years had different NWRNC/100,000 population cut-off points (all children aged < 10 years, 18.7; preschoolers, 13.4; schoolers, 25.9). However, the proportion of SARS-CoV-2 RNA detections over and under the respective cut-off points were identical for the three age groups. The risk ratio (RR) for COVID-19 epidemic-associated SARS-CoV-2 RNA detection in dental wastewater was 5.36 (95% confidence interval [CI], 1.72–16.67; Fisher’s exact test, *p* = 0.0005) (Table 1).

The duration of SARS-CoV-2 RNA positivity (i.e., “detection period”) is important in controlling the risk associated with asymptomatically infected children. We calculated the risk in the “detection period” associated with a COVID-19 outbreak for a total period of 62 weeks (Table 2). Among all children and schoolers aged < 10 years, the NWRNC/100,000 population cut-off points for the detection period were lower than those for “detection” (detection vs. detection period: all children aged < 10 years, 18.7 vs. 12.8; schoolers, 25.9 vs. 20.2), although in preschoolers a similar cut-off point between detection and detection period was observed (13.4 vs. 13.4). Overall, the risk for the COVID-19 epidemic-associated detection period over the cut-off points was higher than that for detection among the three age groups (detection vs. detection period: all children aged < 10 years, 51.9 vs. 78.1%; preschoolers, 51.9 vs. 80.0%; schoolers, 51.9 vs. 80.6%). Below the cut-off points, the risk for the COVID-19 epidemic-associated detection period was not obviously different from that for detection among the three age groups (detection vs. detection period: all children aged < 10 years, 9.7 vs. 10.0%; preschoolers, 9.7 vs. 12.5%; schoolers, 9.7 vs. 9.7%). Compared to detection, the RR for the COVID-19 epidemic-associated detection period increased from 5.36 (95% CI, 1.72–16.67; Fisher’s exact test, *p* = 0.0005) in the three age groups to 7.81 (95% CI, 2.63–23.21; Fisher’s exact test, *p* < 0.0001) in all children aged < 10 years, 6.40 (95% CI, 2.52–16.29; Fisher’s exact test, *p* < 0.0001) in preschoolers, and 8.33 (95% CI, 2.80–24.77; Fisher’s exact test, *p* < 0.0001) in schoolers aged < 10 years.

**Table 2:** Risk of COVID-19 emergence-associated SARS-CoV-2 RNA detection period in wastewater from a pediatric dental clinic in Saitama Prefecture, Japan, by age group.

	NWRNC/100,000 population**	SARS-CoV-2 RNA detection period (total n = 62)***		RR (95% CI); *p* value^†^^†^
		Yes (n = 28)^†^	No (n = 34)	
Total, under 10 Y-olds (n = 571,180)	–	–	–	7.81 (2.63–23.21); *p* < 0.0001
NWRNC* < 73 (n = 30)	< 12.8 (n = 30)	3 (10.0%)	27 (90.0%)	–
≥ 73 (n = 32)	≥ 12.8 (n = 32)	25 (78.1%)	7 (21.9%)	–
Preschoolers (n = 328,311)	–	–	–	6.40 (2.52–16.29); *p* < 0.0001
NWRNC < 44 (n = 32)	< 13.4 (n = 32)	4 (12.5%)	28 (87.5%)	–
≥ 44 (n = 30)	≥ 13.4 (n = 30)	24 (80.0%)	6 (20.0%)	–
Schoolers (n = 242,869)	–	–	–	8.33 (2.80–24.77); *p* < 0.0001
NWRNC < 49 (n = 31)	< 20.2 (n = 31)	3 (9.7%)	28 (90.3%)	–
≥ 49 (n = 31)	≥ 20.2 (n = 31)	25 (80.6%)	6 (19.4%)	–

Data are n (%) or RR (95% CI). RR, risk ratio.

*NWRNC, number of weekly reported new cases.

**The cut-off points of NWRNC per 100,000 population were determined based on ROC curve (Supplementary Table S4).

***The SARS-CoV-2 RNA detection period was 62 weeks in duration.

^†^SARS-CoV-2 RNA detection period was totally 28 weeks (Weeks 29–42 of 2021 and Weeks 8–21 of 2022).

^††^*P*-values obtained by Fisher’s exact test.

## Discussion

Combined with clinical data and contact tracing, wastewater-based epidemiology (WBE) enables monitoring of SARS-CoV-2 community transmission, elucidating the onset, decline, and re-emergence of the epidemic^15^. Compared to the general community wastewater, pediatric dental wastewater can provide a targeted sample of asymptomatic children. This study uniquely detected SARS-CoV-2 RNA in the dental wastewater. The detection of SARS-CoV-2 RNA in 17 samples coincided with COVID-19 emergence among children in Saitama Prefecture. A sample obtained at the end of February 2022 contained the Omicron variant of SARS-CoV-2. Thus, the wastewater of pediatric dental clinics can provide information regarding asymptomatic COVID-19 in children.

Unlike clinical COVID-19 specimens, wastewater samples contain viral RNA at low concentrations. Therefore, we used Ct values as a reference without considering the copy number^16^. To detect SARS-CoV-2, we used the N1 and N2 primers and the probe set developed by the US Centers for Disease Control and Prevention (CDC), which amplify two regions of the nucleocapsid (*N*) gene^17^. In the kit used in this study, CDC N1 and N2 are modified using Cy5 and subjected to PCR in the same tube, thereby doubling the fluorescence intensity and improving assay sensitivity.

In Japan, the age-dependent epidemiological characteristics of COVID-19 varied among epidemic waves^18^. The sixth wave (Omicron variant) differed significantly from previous waves. The proportion of SARS-CoV-2 infections in individuals aged > 70 years was highest during the first wave (variant B.1.1)^19^, with the number of elderly cases gradually decreasing. The number of cases in 20–50-year-olds markedly increased in the second to fifth waves (variants B.1.1.284, B.1.1.214, Alpha, and Delta, respectively)^18,19^. During the sixth wave (Omicron variant)^18^, the number of cases in individuals aged < 19 years increased rapidly.

Regarding vaccination in Japan, the mRNA vaccines developed by Pfizer and Moderna were mainly used^20^. Vaccination using the Pfizer vaccine began in April 2021, while the Moderna vaccine was introduced in May 2021. Initially, the vaccines were available to the elderly, and later the age range for vaccination has been gradually expanded to 12 years and older. Vaccination of children aged 5–11 years started in February 2022. Individuals in the age groups that were not vaccinated may have accounted for the increased number of cases.

Reports from other countries have indicated a high prevalence of the Delta and Omicron variants (particularly the latter) among children, implying that the characteristics of each virus variant were the main determinants of the number of cases^21–23^. In this study of children aged < 10 years in Saitama Prefecture, we observed an indistinct fourth wave that did not fall under the definition of an epidemic wave^18^. During the fifth and sixth waves, the NWRNC/100,000 population similarly increased and decreased for both preschoolers and schoolers aged < 10 years (*p* < 0.0001, Wilcoxon matched-pairs test).

Although this study did not detect SARS-CoV-2 RNA during the period of Alpha-variant dominance, detection began in the same week as that of the fifth wave. Meanwhile, the sixth wave began 5 weeks before SARS-CoV-2 RNA detection. We think that the SARS-CoV-2 RNA detection resulted from asymptomatic patients visiting the hospital. Generally, SARS-CoV-2 excretion begins 2 days before symptom onset and persists for 7–10 days. The incubation period is 1.5 days longer in children than in the elderly^24^, while it is about one day longer for the Delta variant (4.41 days; 95% CI, 3.76–5.05) than for the Omicron variant (3.42 days; 95% CI, 2.88–3.96)^24^. The prolonged incubation period of the Delta variant could have increased the number of hospital visits by asymptomatic patients. Conversely, a significantly greater number of children were affected by the Omicron variant, including those with asymptomatic infections, compared to the Delta variant, and it is estimated that the number of asymptomatic children was much greater during the Omicron-predominant period than during the Delta-predominant period.

**Figure 2:**
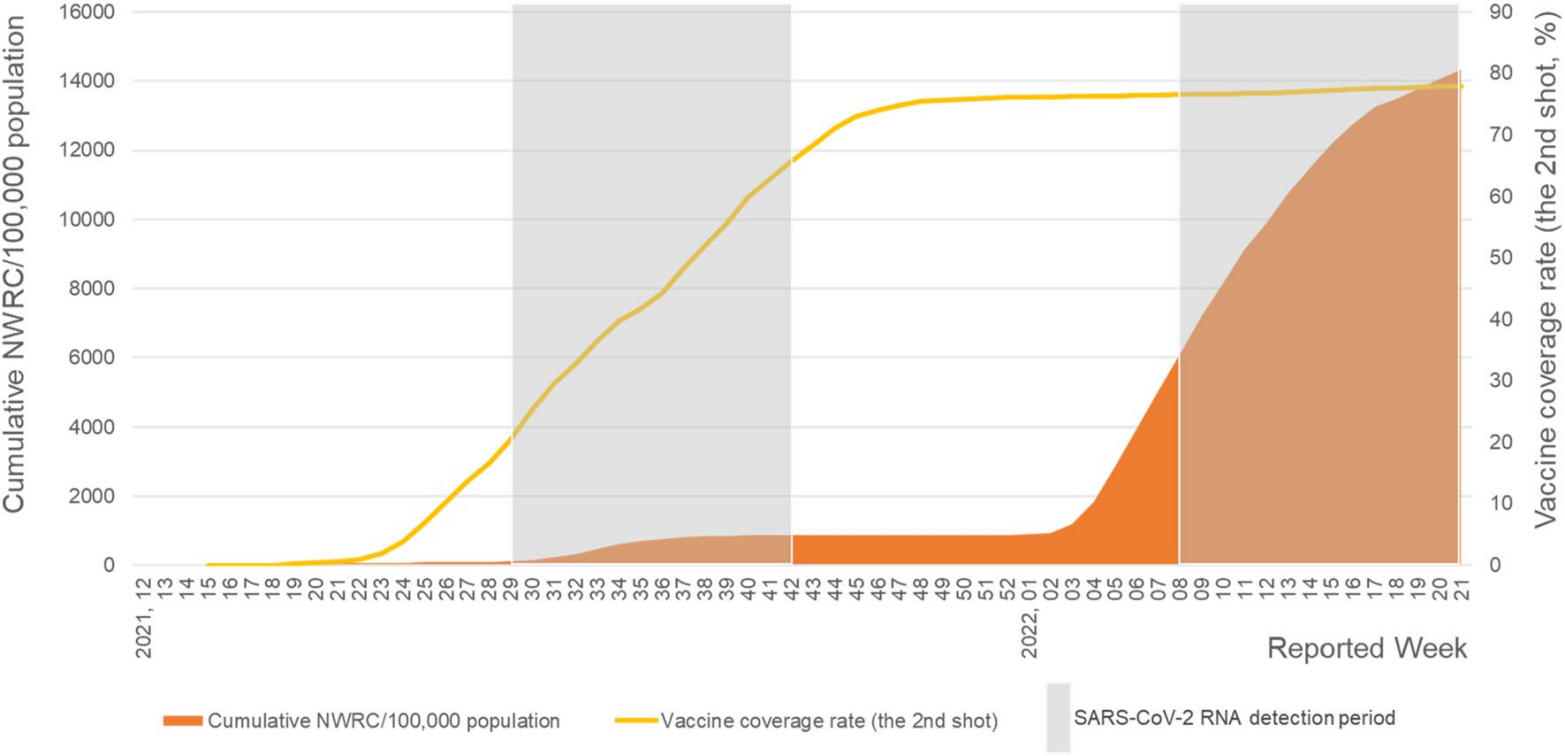
Vaccine coverage rate (the 2nd shot, %)^27^, detection period of SARS-CoV-2 RNA in wastewater from a pediatric dental clinic, and cumulative number of weekly reported new cases (NWRNC) per 100,000 population aged under 10 years in Saitama Prefecture, Japan. The SARS-CoV-2 RNA detection period was observed in Weeks 29–42 of 2021 (the fifth wave, Delta VOC predominant) and Weeks 8–21 of 2022 (the sixth wave, Omicron VOC predominant).

In Japan, COVID-19 vaccination began in April 2021. The vaccination coverage rate (two doses) was approximately 20% during the fifth wave and reached around 75% by December 2021, with over 70% in 12–19-year-olds^20^. While no vaccine was available for children aged < 12 years at the time, the high community vaccination coverage rates could have contributed to the 5-week delay in SARS-CoV-2 RNA detection following the start of the sixth epidemic wave (Fig. 2). The markedly increased NWRNC during the first 5 weeks of the sixth wave may have been associated with increased number of asymptomatic patients visiting the clinic.

In this study, SARS-CoV-2 RNA was detected in wastewater until 3 weeks after the end of the fifth wave, representing a period of high risk in terms of asymptomatic patients visiting the hospital, since COVID-19 infections in children are typically mild or asymptomatic. Additionally, the viral RNA can be detected for 10–14 days after symptom resolution. The number of days required to achieve PCR negativity has been reported to be similar between the Delta and Omicron (BA.1) variants^25^. Long COVID-19 was recognized by the WHO in 2021^26^ and may account for the prolonged period of detection after the fifth wave.

The Omicron variant was detected simultaneously with the initial detection of SARS-CoV-2 RNA during the sixth wave, probably representing the peak of RNA release in this epidemic period. Despite a rapid decline of NWRNC following the peak of the sixth wave, SARS-CoV-2 RNA continued to be detected until the end of the survey period.

In WBE studies, SARS-CoV-2 RNA detection is generally considered a leading indicator of an increase in COVID-19 cases. However, in this study, the Delta and Omicron waves (Weeks 29–39 of 2021 and Weeks 3–21 of 2022, respectively) preceded SARS-CoV-2 RNA detection in dental wastewater (Weeks 29–42 of 2021 and Weeks 8–21 of 2022, respectively). Asymptomatic patients may have visited the clinic as the number of local cases increased. The increase in the number of cases was marginal during the fourth wave (Alpha variant), and SARS-CoV-2 RNA was not detected in wastewater from the pediatric dental clinic. This implies that a threshold number of cases is necessary for SARS-CoV-2 RNA detection in dental wastewater. Receiver operating characteristic (ROC) analysis was used to estimate the cut-off NWRNC/100,000 population value.

The cut-off and risk analysis demonstrated a strong association between SARS-CoV-2 RNA detection in dental wastewater and the NWRNC/100,000 population. The RR, 95% CI, and Fisher’s exact test results were identical among the three groups (RR, 5.36 [95% CI, 1.72–16.67]; Fisher’s exact test, *p* = 0.0005), although the cut-off values differed among the age groups.

During the SARS-CoV-2 RNA detection period, asymptomatic patients may have undergone dental treatment and been in contact with others in the preschool, school, or home settings. For all age groups, the RR was higher in the SARS-CoV-2 RNA detection period compared to that in SARS-CoV-2 RNA detection. Therefore, physicians and public health managers should be aware of the possibility of asymptomatic COVID-19 children when the NWRNC/100,000 population for those aged < 10 years reaches ≥ 12.8 (preschoolers, ≥ 13.4; schoolers aged < 10 years, ≥ 20.2).

SARS-CoV-2 RNA positivity began when the NWRNC among children aged < 10 years reached ≥ 12.8/100,000 population (RR, 7.8 [95% CI, 2.63–23.21]; Fisher’s exact test, *p* < 0.0001). A sample obtained in Week 8 of 2022 was found to harbor the Omicron VOC. This method may also be used to detect other viral pathogens in dental wastewater.

This study had several limitations. Since the data were all from Saitama Prefecture, the findings may not be generalizable to other locations. Therefore, further multicenter studies, using our study as a guide, are needed. Because wastewater samples contain viral RNA at low concentrations, a more comprehensive analysis could be conducted by improving the sensitivity of the assay. Additionally, multivariate analyses are required to overcome the effects of potential confounding factors and bias. To this end, efforts to gather more data for multivariate models, conduct several multicenter studies, and sensitivity improvement trials are currently underway.

## Conclusions

The number of COVID-19 cases among individuals < 10 years of age was associated with the SARS-CoV-2 RNA positivity in wastewater from a pediatric dental clinic. Since wastewater from pediatric dental clinics originates from the oral cavities of asymptomatic children, such testing can provide important information regarding asymptomatic COVID-19 in children, complementing clinical pediatric data.

## Methods

### Study design

This was a prospective longitudinal observational study conducted at the Meikai University Hospital, the only dental hospital in Saitama Prefecture, located at its center (Supplementary Fig. S1). Meikai University Hospital has several medical and dental departments, including oral diagnostics, pediatric dentistry, oral surgery, orthodontics, oral health/sports dentistry, conservative and restorative dentistry, periodontics, endodontics, and prosthodontics. The pediatric dental clinic offers restorative and preventive treatment for children, from before the eruption of baby teeth until the eruption of permanent teeth. Most patients presenting to the pediatric dental clinic are residents of Saitama Prefecture aged < 10 years. To investigate the effect of local community infection status (exposure; NWRNC/100,000 population) on wastewater SARS-CoV-2 RNA detection in a pediatric dental clinic (outcome), we compared the NWRNC per 100,000 population with the SARS-CoV-2 RNA detection results.

The exclusion criteria for this study were (1) symptomatic COVID-19, (2) close contact with a symptomatic COVID-19 patient, (3) class closure due to a cluster outbreak at school or preschool, (4) at least one cold symptom (fever, cough, headache, runny nose, or shortness of breath), and (5) alterations or loss of taste/smell. Inclusion criteria were children with none of the above five exclusion criteria (1) to (5).

We planned to obtain a total of 60 samples from the pediatric dental clinic each Saturday from Week 11 of 2021 to Week 21 of 2022 (i.e., from 20 March 2021 to 28 May 2022, Supplementary Table S3). Weeks 32 and 52 of 2021 were holidays at the Meikai University Hospital, and Week 7 of 2022 was students’ clinical exam week. Therefore, sampling was not performed during these weeks. The COVID-19 pandemic in Japan lasted from January 2020 to March 2021 (1 year and 3 months [64 weeks]) and comprised three waves. At baseline, we expected the three waves to occur within the sample collection period. Moreover, based on the sample size calculation for Fisher’s exact test (discussed below), a sample size of 60 was considered adequate.

### Dental wastewater collection

Since SARS-CoV-2 has a propensity to adhere to solid surfaces^27^, samples were obtained from the plastic drainage filters of the dental chairs. Each dental chair had one drainage filter for wastewater, with samples thus being completely isolated from other sections of the hospital. Since the drainage filters at the pediatric dental clinic were cleaned every Saturday using 0.05% sodium hypochlorite, we scheduled wastewater sampling prior to cleaning. We hypothesized that the samples were representative of the asymptomatic COVID-19 patients treated each week at the pediatric dental clinic. The plastic drainage filters were soaked in 500 mL of Dulbecco’s phosphate-buffered saline, and viral RNA was diffused into the buffer using an ultrasonic cleaner for 1 min. The virus was inactivated by incubating the samples in a water bath at 56 °C for 40 min^16,28^, and the samples were stored at − 80 °C.

### Viral RNA concentration, extraction, and purification

Viral RNA was concentrated using the polyethylene glycol (PEG) precipitation method. After adding NaCl and PEG, the samples were centrifuged for 99 min^29^ instead of shaking overnight^16^. The QIAamp Viral RNA Mini Kit (Qiagen, Hilden, Germany) was used for viral RNA extraction and purification^16^.

### Real-time reverse transcription PCR (RT-PCR)

SARS-CoV-2 RNA was detected using the SARS-CoV-2 Detection RT-qPCR Kit for Wastewater (Takara Bio, Shiga, Japan) and QuantStudio 5 Real-Time PCR System (Thermo Fisher Scientific, Waltham, MA, USA). The primers and probes are listed in Supplementary Table S5. The reaction conditions were based on the manufacturers’ instructions. The threshold relative fluorescence intensity (ΔRn) and cycle threshold (Ct) values were determined using the QuantStudio 5 Real-Time PCR System (Thermo Fisher Scientific) analysis software. An amplified result within 40 Ct was considered positive, as recommended by the manufacturer. If both positive and negative results were obtained from a sample, indicating the presence of viral genes at very low concentrations, the sample was considered positive.

As a process control, real-time RT-PCR was performed for the human ribonuclease (RNase) P gene (GenBank accession number NM_006413) using the primers and probe described previously (Supplementary Table S5)^30^. The detection kit included a primer and probe for pepper mild mottle virus (PMMoV) as the process control^16^. Although the PMMoV concentration in general community wastewater was high^16,31,32^, the dental wastewater did not contain any feces, which is the major source of PMMoV. RNase P has previously been used as a process control for COVID-19 patient samples^17,30^. The following synthetic DNAs were used as positive controls for RNase P (Hokkaido System Science, Hokkaido, Japan): RP-DNA, GCAGATTTGGACCTGCGAGCGGGTTCTGACCTGAAGGCTCTGCGCGGACTTGTGGAGACAGCCGCTCAC.

For SARS-CoV-2 RNA-positive samples, the N501Y, L452R, and G339D mutations were detected using the SARS-CoV-2 Direct Detection RT-qPCR Core Kit with the primers/probes N501Y (SARS-CoV-2), L452R (SARS-CoV-2) Ver. 2, and G339D (SARS-CoV-2) (Takara Bio), under reaction conditions based on the manufacturer’s instructions. Positive control RNAs were obtained from Takara Bio.

### Regional infected pediatric populations

Weekly COVID-19 prevalence reports for < 10-year-old preschoolers and schoolers from Week 12 of 2021 to Week 21 of 2022 (sampling failed in Week 11 of 2021) were obtained from the government of Saitama Prefecture^33^. Data for the Saitama Prefecture population were obtained from the government of Saitama Prefecture’s website^34^. At baseline, we expected multiple epidemic waves within the surveillance period. An epidemic wave was defined as follows: onset, increase in prevalence for ≥ 3 weeks and NWRNC ≥ 10% of the peak; termination, decrease in prevalence for ≥ 3 weeks and NWRNC < 10% of the peak according to the National Institute of Infectious Diseases, Japan^18^.

### Statistical analysis

We used JMP software (v. 14.3; SAS Institute, Cary, NC, USA) for the analysis. We compared NWRNC between preschoolers and schoolers aged < 10 years using the Wilcoxon matched-pairs test and Spearman’s correlation.

To assess the associations between the NWRNC per 100,000 population in Saitama Prefecture (exposure) and SARS-CoV-2 RNA positivity in wastewater from the pediatric dental clinic (outcome), the RR and 95% CI were calculated, and Fisher’s exact test was performed. Since the outcome was a categorical variable and the exposure was a continuous variable, through ROC curve analysis, the area under the ROC curve (AUC)^35,36^ was calculated to obtain the optimal cut-off value, then a 2-by-2 analysis was performed.

The sample size for Fisher’s exact test^37,38^was calculated on the basis of the following hypotheses:

- Null hypothesis (${H}_{0}$): population variance (${\sigma }^{2}$) is equal to the specified sample variance (${\sigma }_{0}^{2}$).
- Alternative hypothesis (${H}_{1}$): population variance (${\sigma }^{2}$) is not equal to the specified sample variance (${\sigma }_{0}^{2}$).
- The approximate sample size ($n$) was estimated using the following equation:
  $$n \approx \frac{1}{2} {\left(\frac{{Z}_{\alpha /2}-\Delta {Z}_{1-\beta }}{\Delta -1}\right)}^{2}+\frac{3}{2}$$

where ${\sigma }^{2}$ represents the population variance, ${\sigma }_{0}^{2}$ represents the specified sample variance, $\Delta = \frac{\sigma }{{\sigma }_{0}}$ is the ratio of population variance to specified sample variance, $Z$ is a variable following the ${\chi }^{2}$ distribution, approximated by a normal distribution in the context of Fisher’s exact test, $\alpha$ is the significance level when determining the probability of rejecting the null hypothesis when it is true, and $1-\beta$ is the power of the test, representing the probability of correctly rejecting the null hypothesis.

### Ethical approval

The study protocol was reviewed and approved by the Institutional Review Board of Meikai University School of Dentistry (IRB #A2029). The need for written informed consent was waived because the samples were not linked to any individuals. All procedures were performed in accordance with relevant guidelines and regulations.

### Supplementary Information

Supplementary Information.

## Data Availability

Data are contained within the article or Supplementary Materials.

## References

[1] Centers for Disease Control and Prevention. *Pediatric Data*, <https://covid.cdc.gov/covid-data-tracker/#pediatric-data> (2023).

[2] Woodruff RC (2022). Risk factors for severe COVID-19 in children. Pediatrics.

[3] Godfred-Cato S (2021). Multisystem inflammatory syndrome in infants <12 months of age, United States, May 2020-January 2021. Pediatr. Infect. Dis. J..

[4] Prime Minister's office of Japan. *Press Conference by the Prime Minister regarding the Discussion toward Reclassifying COVID-19 as a Class V Infectious Disease*, <https://japan.kantei.go.jp/101_kishida/statement/202301/_00008.html> (2023).

[5] Bivins A (2020). Wastewater-based epidemiology: Global collaborative to maximize contributions in the fight against COVID-19. Environ. Sci. Technol..

[6] Miyazawa S (2024). Wastewater-based reproduction numbers and projections of COVID-19 cases in three areas in Japan, November 2021 to December 2022. Euro. Surveill..

[7] Kagami K, Kitajima M, Takahashi H, Teshima T, Ishiguro N (2023). Association of wastewater SARS-CoV-2 load with confirmed COVID-19 cases at a university hospital in Sapporo, Japan during the period from February 2021 to February 2023. Sci. Total Environ..

[8] Ando H (2023). Wastewater-based prediction of COVID-19 cases using a highly sensitive SARS-CoV-2 RNA detection method combined with mathematical modeling. Environ. Int..

[9] Tanimoto Y (2022). SARS-CoV-2 RNA in wastewater was highly correlated with the number of COVID-19 cases during the fourth and fifth pandemic wave in Kobe City, Japan. Front. Microbiol..

[10] Hata A, Hara-Yamamura H, Meuchi Y, Imai S, Honda R (2021). Detection of SARS-CoV-2 in wastewater in Japan during a COVID-19 outbreak. Sci. Total Environ..

[11] To KK (2020). Consistent detection of 2019 novel coronavirus in saliva. Clin. Infect. Dis..

[12] Castro-Gutierrez V (2022). Monitoring occurrence of SARS-CoV-2 in school populations: A wastewater-based approach. PLoS One.

[13] Wolken M (2023). Wastewater surveillance of SARS-CoV-2 and influenza in preK-12 schools shows school, community, and citywide infections. Water Res..

[14] Conway DI (2021). SARS-CoV-2 positivity in asymptomatic-screened dental patients. J. Dent. Res..

[15] Li X (2023). Correlation between SARS-CoV-2 RNA concentration in wastewater and COVID-19 cases in community: A systematic review and meta-analysis. J. Hazard Mater..

[16] COVID-19 Taskforce. *Manual for detection of SARS-CoV-2 RNA in wastewater*, <chrome-extension://efaidnbmnnnibpcajpcglclefindmkaj/https://www.jswe.or.jp/aboutus/pdf/Manual-for-Detection-of-SARS-CoV-2-RNA-in-Wastewater.pdf> (2022).

[17] Centers for Disease Control and Prevention (CDC). *2019-novel coronavirus (2019-nCoV) real-time rRT-PCR panel primers and probes*, <https://stacks.cdc.gov/view/cdc/84525/cdc_84525_DS1.pdf> (2020).

[18] Otani, K. T., Ko, Y., Yamauchi, Y., Suzuki, M. Epidemiological study of gender and age characteristics for each epidemic wave of novel coronavirus infection in Japan. Report No. 514, 271–272 (National Institute of infectious diseases, Tokyo, 2022).

[19] Tsuyoshi Sekizuka, K. I., Yatsu, K., Tanaka, R., Eto, S., Someno, R., Hashino, M., Kuroda, M., COVID-19 Genome Surveillance Group. Molecular epidemiological survey using novel coronavirus SARS-CoV-2 genome information (as of January 14, 2021). Report No. 493, 13–14 (National Instiyute of Infectious Diseases, Tokyo, 2021).

[20] National Institute of Infectious Diseases. *About the new coronavirus (as of December 16, 2021)*, <https://www.niid.go.jp/niid/ja/2019-ncov/2484-idsc/10840-covid19-64.html> (2021).

[21] Chiwandire N (2023). Changing epidemiology of COVID-19 in children and adolescents over four successive epidemic waves in South Africa, 2020–2022. J. Pediatric. Infect. Dis. Soc..

[22] Hamid, S. *et al.* COVID-19-associated hospitalizations among U.S. infants aged <6 months - COVID-NET, 13 states, June 2021-August 2022. *MMWR Morb. Mortal. Wkly. Rep.* **71**, 1442–1448. 10.1558/mmwr.mm7145a3 (2022). 10.15585/mmwr.mm7145a3 PMC9707352 36355608

[23] Chun JY, Jeong H, Kim Y (2022). Identifying susceptibility of children and adolescents to the Omicron variant (B.1.1.529). BMC Med..

[24] Wu Y (2022). Incubation period of COVID-19 caused by unique SARS-CoV-2 strains: A systematic review and meta-analysis. JAMA Netw. Open.

[25] Boucau J (2022). Duration of shedding of culturable virus in SARS-CoV-2 Omicron (BA.1) infection. N. Engl. J. Med..

[26] Soriano JB (2022). A clinical case definition of post-COVID-19 condition by a Delphi consensus. Lancet Infect. Dis..

[27] Dehbandi R, Zazouli MA (2020). Stability of SARS-CoV-2 in different environmental conditions. Lancet Microbe.

[28] Pastorino B, Touret F, Gilles M, de Lamballerie X, Charrel RN (2020). Heat inactivation of different types of SARS-CoV-2 samples: What protocols for biosafety, molecular detection and serological diagnostics?. Viruses.

[29] IDEXX laboratories. *Sample Concentration Protocol for Wastewater Surveillance for SARS-CoV-2*, <https://www.idexx.com/files/example-concentration-protocol-for-wastewater-surveillance-PEG.pdf> (2020).

[30] Emery SL (2004). Real-time reverse transcription-polymerase chain reaction assay for SARS-associated coronavirus. Emerg. Infect. Dis..

[31] Haramoto E (2018). A review on recent progress in the detection methods and prevalence of human enteric viruses in water. Water Res..

[32] Alamin M, Tsuji S, Hata A, Hara-Yamamura H, Honda R (2022). Selection of surrogate viruses for process control in detection of SARS-CoV-2 in wastewater. Sci. Total. Environ..

[33] Saitama Prefecture. *Status of new coronavirus infections in Saitama Prefecture*, <https://www.pref.saitama.lg.jp/a0701/covid19/jokyo.html> (2023).

[34] Saitama Prefecture. *Statistics in Saitama Prefecture*, <https://www.pref.saitama.lg.jp/a0206/a009/r03age.html> (2023).

[35] Nahm FS (2022). Receiver operating characteristic curve: Overview and practical use for clinicians. Korean J. Anesthesiol..

[36] Hanley JA, McNeil BJ (1982). The meaning and use of the area under a receiver operating characteristic (ROC) curve. Radiology.

[37] Fisher RA (1922). On the Interpretation of χ2 from contingency tables, and the calculation of P. J. Royal Stat. Soc..

[38] Nagata, Y. *How to determine sample size*. Vol. 1 (Asakura Bookstore, 2003).

