# Supplementary material for: SARS-CoV-2 detection in pediatric dental clinic wastewater reflects the number of local COVID-19 cases in children under 10 years old
Source: Sci Rep. 2024 May 28;14:12187. doi: 10.1038/s41598-024-63020-z (PMC11133353; doi:10.1038/s41598-024-63020-z)
Supplement: Supplementary file 1 — Supplementary Information. [file 41598_2024_63020_MOESM1_ESM.pdf]

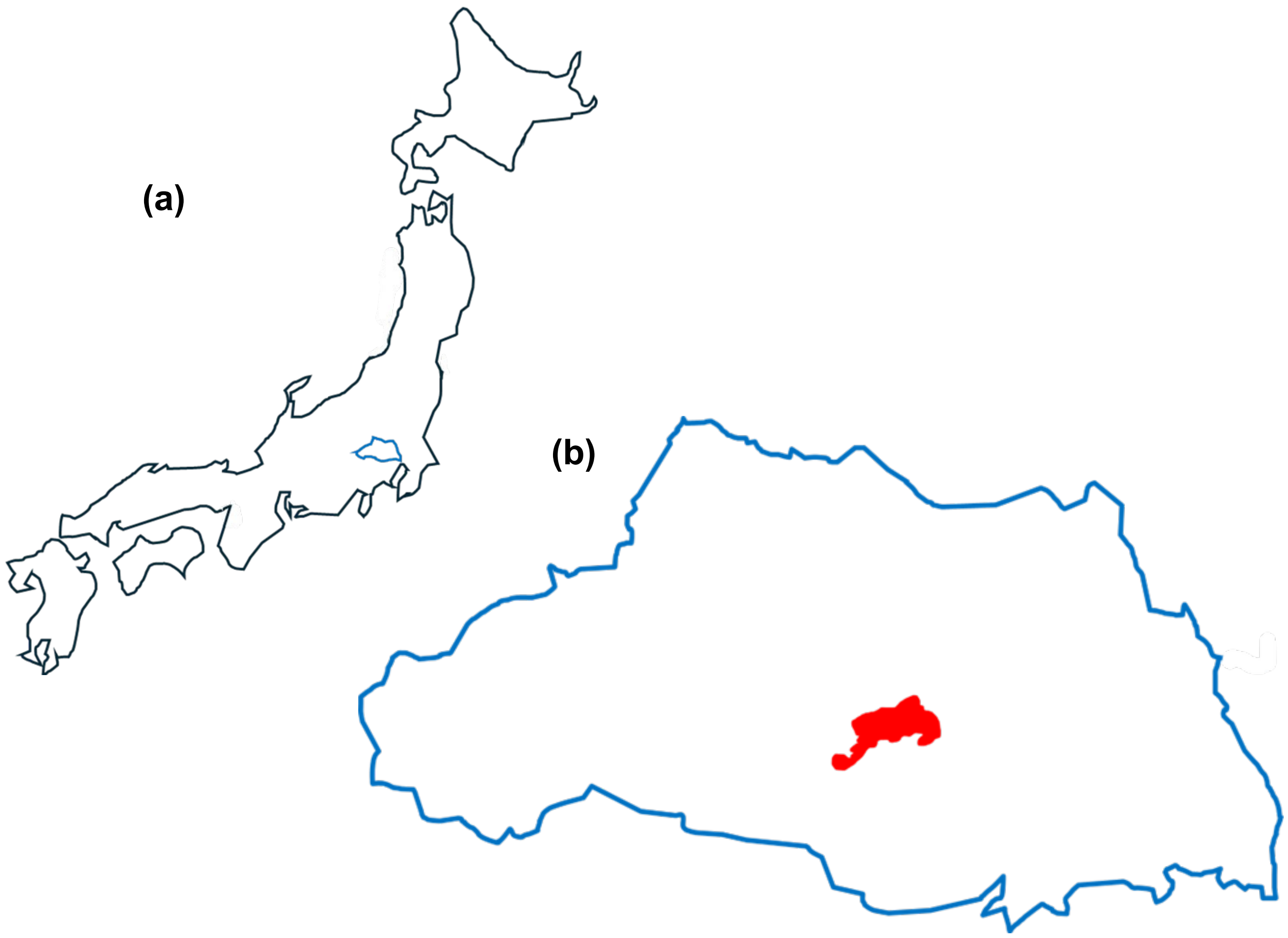

Figure S1. Japan (a) and Saitama prefecture (b).
Blue-outlined, Saitama Prefecture; Red-highlighted, Sakado city.
This graphic was made by Microsoft 365 PowerPoint 2404
(https://www.microsoft.com/ja-jp/microsoft-365/powerpoint)

Table S1-1. Detection results of SARS-CoV-2 RNA from dental wastewater and number of weekly reported new cases (NWRNC) under 10-year-old, Saitama prefecture, Japan.

Year & Week no.	Detection from dental wastewater [a]	NWRNC		
		Total, under 10 Y-olds (n=571,180)	Preschoolers (n=328,311)	Schoolers (n=242,869)
2021, 12	−	32	12	20
13	−	32	17	15
14	−	34	13	21
15	−	38	14	24
16	−	56	18	38
17	−	47	13	34
18	−	44	11	33
19	−	11	3	8
20	−	61	30	31
21	−	33	17	16
22	−	16	9	7
23	−	22	5	17
24	−	19	3	16
25	−	17	5	12
26	−	11	0	11
27	−	56	19	37
28	−	77	37	40
29	+	107	44	63
30	+	201	75	126
31	+	422	147	275
32	ND [b]	575	189	386
33	+	740	273	467
34	+	803	330	473
35	−	546	207	339
36	+	418	156	262
37	−	268	121	147
38	−	146	51	95
39	−	73	24	49
40	+	30	11	19
41	+	10	6	4
42	+	8	5	3
43	−	7	3	4
44	−	11	8	3
45	−	4	4	0
46	−	5	1	4
47	−	8	0	8
48	−	10	5	5
49	−	5	3	2
50	−	5	3	2
51	−	9	5	4
52	ND [b]	18	12	6
2022, 01	−	47	14	33
02	−	313	97	216
03	−	1392	438	954
04	−	3637	1252	2385
05	−	5931	1961	3970
06	−	6233	2200	4033
07	ND [c]	6296	2306	3990
08	+ [d]	6197	2304	3893
09	−	6127	2299	3828
10	+	5459	2038	3421
11	−	5435	2001	3434
12	+	4391	1558	2833
13	ND [e]	4872	1686	3186
14	−	4064	1512	2552
15	+	4224	1596	2628
16	+	3243	1162	2081
17	+	2800	931	1869
18	−	1348	370	978
19	−	1603	551	1052
20	+	1743	643	1100
21	+	1390	459	931
	Cumulative number	81780	29287	52493

[a] −, negative; +, positive

[b] week no.32 and 52 in 2021 were holiday weeks in the Meikai University Hospital.

[c] week no.07 in 2022 was a students' clinical examination week in the Meikai University Hospital.

[d] week 08 in 2022, a mutation, G339D on spike protein of SARS-CoV-2 was detected using a commercial kit (SARS-CoV-2 Direct Detection RT-qPCR Core Kit with Primer/Probe G339D (SARS-CoV-2) (TAKARA, Shiga, Japan)). Ct = 37.8.

[e] sample at the week 13 in 2022 was not determined, because process control (RNase P) was not detected.

Table S1-2. Detection results of SARS-CoV-2 RNA from dental wastewater and number of weekly reported new cases (NWRNC) /100,000 population under 10-year-old, Saitama prefecture, Japan.

Year & Week no.	Detection from dental wastewater [a]	NWRNC/100,000 population		
		Total, under 10 Y-olds (n=571,180)	Preschoolers (n=328,311)	Schoolers (n=242,869)
2021, 12	−	5.6	3.7	8.2
13	−	5.6	5.2	6.2
14	−	6	4.0	8.6
15	−	6.7	4.3	9.9
16	−	9.8	5.5	15.6
17	−	8.2	4.0	14
18	−	7.7	3.4	13.6
19	−	1.9	0.9	3.3
20	−	10.7	9.1	12.8
21	−	5.8	5.2	6.6
22	−	2.8	2.7	2.9
23	−	3.9	1.5	7
24	−	3.3	0.9	6.6
25	−	3	1.5	4.9
26	−	1.9	0.0	4.5
27	−	9.8	5.8	15.2
28	−	13.5	11.3	16.5
29	+	18.7	13.4	25.9
30	+	35.2	22.8	51.9
31	+	73.9	44.8	113.2
32	ND [b]	100.7	57.6	158.9
33	+	129.6	83.2	192.3
34	+	140.6	100.5	194.8
35	−	95.6	63.0	139.6
36	+	73.2	47.5	107.9
37	−	46.9	36.9	60.5
38	−	25.6	15.5	39.1
39	−	12.8	7.3	20.2
40	+	5.3	3.4	7.8
41	+	1.8	1.8	1.6
42	+	1.4	1.5	1.2
43	−	1.2	0.9	1.6
44	−	1.9	2.4	1.2
45	−	0.7	1.2	0
46	−	0.9	0.3	1.6
47	−	1.4	0.0	3.3
48	−	1.8	1.5	2.1
49	−	0.9	0.9	0.8
50	−	0.9	0.9	0.8
51	−	1.6	1.5	1.6
52	ND [b]	3.2	3.7	2.5
2022, 01	−	8.2	4.3	13.6
02	−	54.8	29.5	88.9
03	−	243.7	133.4	392.8
04	−	636.8	381.3	982
05	−	1038.4	597.3	1634.6
06	−	1091.2	670.1	1660.6
07	ND [c]	1102.3	702.4	1642.9
08	+ [d]	1084.9	701.8	1602.9
09	−	1072.7	700.3	1576.2
10	+	955.7	620.8	1408.6
11	−	951.5	609.5	1413.9
12	+	768.8	474.6	1166.5
13	ND [e]	853	513.5	1311.8
14	−	711.5	460.5	1050.8
15	+	739.5	486.1	1082.1
16	+	567.8	353.9	856.8
17	+	490.2	283.6	769.6
18	−	236	112.7	402.7
19	−	280.6	167.8	433.2
20	+	305.2	195.9	452.9
21	+	243.4	139.8	383.3
Cumulative number		14317.7	8920.5	21613.7

[a] −, negative; +, positive
[b] week 32 and 52 in 2021 were holiday weeks in the Meikai University Hospital.
[c] week 07 in 2022 was a students' clinical examination week in the Meikai University Hospital.
[d] week 08 in 2022, a mutation, G339D on spike protein of SARS-CoV-2 was detected using a commercial kit (SARS-CoV-2 Direct Detection RT-qPCR Core Kit with Primer/Probe G339D (SARS-CoV-2) (TAKARA)). Ct = 37.8.
[e] sample at the week 13 in 2022 was not determined, because process control (RNase P) was not detected.

Table S2. The number of patients at the pediatric dental clinic in Meikai University Hospital, Saitama, Japan.

Year	Month	No. of Patients
2021	March[a]	188 [a]
2021	April	763
2021	May	587
2021	June	672
2021	July	791
2021	August	809
2021	September	691
2021	October	668
2021	November	576
2021	December	731
2022	January	619
2022	February	570
2022	March	779
2022	April	733
2022	May	512
Total	14.25 months	9689

[a] Wastewater sampling was started from week 11, 2021. However, the fist sampling was failed, thus we calculated from week 12, 2027 (as a quarter number of that in March).

Table S3. Detection results and Ct value of SARS-CoV-2 from dental wastewater.

Year & Week no.	SARS-CoV-2			RNase-P [e]	
	Well 1	Well 2	detection	Well 1	Well 2
2021, 11	ND [a]	ND	ND	ND	ND
12	−	−	−	30.6	30.7
13	−	−	−	31.7	31.4
14	−	−	−	33.5	32.6
15	−	−	−	30.7	30.9
16	−	−	−	32.4	31.4
17	−	−	−	33.4	34.8
18	−	−	−	32.4	32.7
19	−	−	−	31.7	32.2
20	−	−	−	30.8	31.5
21	−	−	−	32.3	32.0
22	−	−	−	31.0	31.3
23	−	−	−	30.1	29.9
24	−	−	−	30.8	31.2
25	−	−	−	31.5	31.9
26	−	−	−	30.1	30.2
27	−	−	−	33.7	33.6
28	−	−	−	32.1	31.5
29	−	37.1	+	31.5	31.6
30	36.8	30.3	+	32.2	32.3
31	31.0	30.8	+	31.9	30.9
32	ND [b]	ND	ND	ND	ND
33	−	31.6	+	31.1	30.4
34	33.2	−	+	33.2	32.8
35	−	−	−	33.4	34.0
36	−	29.2	+	30.2	30.2
37	−	−	−	31.3	30.9
38	−	−	−	32.8	33.0
39	−	−	−	31.5	31.4
40	29.1	−	+	31.4	31.6
41	43.8	29.3	+	32.8	32.6
42	34.4	−	+	33.5	33.8
43	−	−	−	32.5	32.5
44	−	−	−	32.7	32.5
45	−	−	−	33.3	33.7
46	−	−	−	31.5	31.4
47	−	−	−	33.1	32.5
48	−	−	−	33.8	34.7
49	−	−	−	32.8	33.2
50	−	−	−	32.6	32.6
51	−	−	−	33.2	32.7
52	ND [b]	ND	ND	ND	ND
2022, 01	−	−	−	31.4	31.3
02	−	−	−	32.1	31.9
03	−	−	−	31.6	31.9
04	−	−	−	32.7	32.8
05	−	−	−	30.2	30.3
06	−	−	−	32.6	32.5
07	ND [c]	ND	ND	ND	ND
08	34.1	35.5	+ [d]	31.0	30.7
09	−	−	−	31.3	31.3
10	32.8	−	+	28.0	28.1
11	−	−	−	29.7	29.6
12	37.4	−	+	32.4	32.4
13	ND [a]	ND	ND	−	−
14	−	−	−	30.8	30.4
15	−	34.8	+	34.5	34.8
16	37.4	−	+	33.1	33.4
17	−	31.9	+	34.5	34.7
18	−	−	−	36.8	36.7
19	−	−	−	36.6	35.8
20	33.5	−	+	31.9	37.4
21	27.9	30.1	+	32.9	33.8

[a] Sample at the week 11 in 2021 was not determined, because the sampling was failed. Sample at the week 13 in 2022 was not determined, because process control (RNase P) was not detected.
[b] Week 32 and 52 in 2021 were holiday weeks in the Meikai University Hospital.
[c] Week 07 in 2022 was a students' clinical examination week in the Meikai University Hospital.
[d] Week 08 in 2022, a mutation, G339D on spike protein of SARS-CoV-2 was detected using a commercial kit (SARS-CoV-2 Direct Detection RT-qPCR Core Kit with Primer/Probe G339D (SARS-CoV-2) (TAKARA, Shiga, Japan)). Ct = 37.8.
[e] Process control; To monitor PCR inhibitors, the loss of viral particle/genome within the procedures, and nucleic acid extraction efficiency.

Table S4-1. the cut-off points for NWRNC per 100,000 population in Table 1 (Risk of COVID-19 emergence-associated SARS-CoV-2 RNA detection in wastewater from a pediatric dental clinic in Saitama Prefecture, Japan, by age group) and the AUC with 95% confidence interval (CI) that determined the cut-off point.

	Cut-off point		AUC (95% CI)
	NWRNC*	NWRNC/100,000 population	
Total, under 10 Y-olds	107	18.7	0.72 (0.57-0.87)
Preschoolers	44	13.4	0.74 (0.59-0.89)
Schoolers	63	25.9	0.71 (0.55-0.87)

* NWRNC, number of weekly reported new cases

Table S4-2. the cut-off points for NWRNC per 100,000 population in Table 2 (Risk of COVID-19 emergence-associated SARS-CoV-2 RNA detection period in wastewater from a pediatric dental clinic in Saitama Prefecture, Japan, by age group) and the AUC with 95% confidence interval (CI) that determined the cut-off point.

	Cut-off point		AUC (95% CI)
	NWRNC*	NWRNC/100,000 population	
Total, under 10 Y-olds	73	12.8	0.81 (0.70-0.92)
Preschoolers	44	13.4	0.83 (0.72-0.93)
Schoolers	49	20.2	0.81 (0.70-0.92)

* NWRNC, number of weekly reported new cases

Table S5. Primers and probes used in this study.

Assay	Primer and probe name	Sequence (5' to 3')	Concentration
CDC N1 [a]	2019-nCoV_N1-F	GACCCCAAAATCAGCGAAAT	Unknown [a]
	2019-nCoV_N1-R	TCTGGTTACTGCCAGTTGAATCTG	
	2019-nCoV_N1-P	Cy5-ACCCCGCATTACGTTTGGTGGACC-BHQ1	
CDC N2 [a]	2019-nCoV_N2-F	TTACAAACATTGGCCGCAAA	
	2019-nCoV_N2-R	GCGCGACATTCCGAAGAA	
	2019-nCoV_N2-P	Cy5-ACAATTTGCCCCCAGCGCTTCAG-BHQ1	
RNase P [b]	RNase_P-F	AGATTTGGACCTGCGAGCG	375nM
	RNase_P-R	GAGCGGCTGTCTCCACAAGT	375nM
	RNase_P-P	FAM-TTCTGACCTGAAGGCTCTGCGCG-BHQ1	125nM

[a] CDC, 2020[15], including the SARS-CoV-2 Detection RT-qPCR Kit for Wastewater (TAKARA, Shiga, Japan); [b] Emery SL, et al., 2004[12].
